# Provitamin D_3_ modulation through prebiotics supplementation: simulation based assessment

**DOI:** 10.1038/s41598-019-55699-2

**Published:** 2019-12-17

**Authors:** Sucheta Gokhale, Anirban Bhaduri

**Affiliations:** Innovation Center, Tata Chemicals Ltd, Ambedveth, Pune, Maharashtra 412111 India

**Keywords:** Biochemical reaction networks, Nutrition

## Abstract

Vitamin D is important in multiple health conditions. Vitamin D deficiency is prevalent globally even with exposure to adequate sunlight. Reduction in provitamin D_3_ (7-dehydrocholesterol, 7-DHC) is an important cause of vitamin D_3_ deficiency. Vitamin supplementation, food fortification, and use of probiotics are some approaches to reduce vitamin D_3_ deficiency. This study investigates plausibility of 7-DHC biosynthesis through dietary prebiotics supplementation. Furthermore, it reports mechanistic details and constraints for the biosynthesis using flux balance analysis (FBA) simulations. The FBA simulations using co-metabolism models comprising human host and a resident bacterium (*Faecalibacterium prausnitzii* or *Bacteroides thetaiotamicron*) indicated increased flux of 7-DHC with short-chain fructooligosaccharide (scFOS) or inulin supplementation. We observed around 2-fold increase in flux compared to the baseline. Biosynthesis of 7-DHC was primarily modulated through acetate, pyruvate and lactate secreted by the bacterium. We observed diverse mechanisms and dose dependent responses. We extended this assessment to 119 resident bacteria and investigated the metabolites profiles with prebiotics supplementation. In summary, the current study suggests the potential use of applying prebiotics in enhancing 7-DHC biosynthesis. Furthermore, performance of the different gut bacteria with prebiotic supplementation for secreted metabolites profile is reported. These results may be useful to design future clinical studies.

## Introduction

Vitamin D is important in multiple health conditions such as rickets, osteoporosis, muscle weakness, autoimmune disorders, type 1 diabetes, cardiovascular diseases, tuberculosis, and depression^1^. Though being important in a wide spectrum of health conditions, vitamin D deficiency is prevalent globally^2^ and hence a major concern for public health. It was estimated that around 1 billion people globally have either insufficiency or deficiency of vitamin D^3^.

Vitamin D, essentially a fat soluble prohormone steroid, is present in two forms viz., vitamin D_2_ (ergocalciferol) and vitamin D_3_ (cholecalciferol). Vitamin D_2_ is present in few food sources such as sun dried yeast or mushrooms, while vitamin D_3_ can be obtained from animal sources such as fatty fish, and milk. Additionally, vitamin D_3_ is synthesized from its precursor 7-dehydrocholesterol (7-DHC or provitamin D_3_) present in human skin on adequate exposure to UVB radiation^4^.

Many extrinsic and intrinsic factors such as latitude, use of sunscreen, covered clothing habits, age, and skin melanin content are known to lead to vitamin D deficiency^5^. Extrinsic causes such as use of sunscreen^6^, latitude, and covered clothing habits lead to restricted exposure to UVB radiation and result in vitamin D_3_ deficiency^7^. However, it should be noted that, in spite of having sufficient exposure to sunlight, global populations having vitamin D_3_ deficiency are known. Age and skin melanin content are noteworthy intrinsic factors affecting 7-DHC dependent cutaneous synthesis of vitamin D_3_. It is observed that there is a marked age dependent decrease in 7-DHC levels leading to reduced synthesis of vitamin D_3_^1,8^. The study by Holick and coworkers has shown that there is around 75% reduction in cutaneous 7-DHC levels resulting in proportionate decrease in vitamin D_3_ synthesis even with the exposure to sunlight^9^. Another factor leading to reduced biosynthesis of vitamin D_3_ is the skin melanin content. Melanin pigment competes with 7-DHC for UVB absorption. Thus, higher melanin content results in reduced cutaneous synthesis of vitamin D_3_^5,10^. In addition to these factors, post burn scar tissue has been shown to contain around 40% less 7-DHC and in the absence of supplementation such patients developed vitamin D deficiency^7,11^. This indicates that similar to limited sunlight exposure, reduction of 7-DHC is another crucial factor contributing to vitamin D_3_ deficiency. In the current study, we aimed to address vitamin D_3_ deficiency by increasing biosynthesis of its precursor 7-DHC.

Being a public health concern multiple approaches have been used to reduce vitamin D_3_ deficiency such as vitamin supplementation, fortified food, and UVB exposure^12^. However, only supplementation of vitamin D may not work in case of disorders resulting in poor intestinal absorption^13,14^. Recently probiotics have been shown to be effective supplements for reducing vitamin D deficiency. Studies have reported that probiotics show enhanced vitamin D absorption, increased VDR expression, and have multiple related health benefits^15,16^. Interestingly, a clinical study has reported significant increase in serum 25-hydroxyvitamin D_3_ levels with oral supplementation of probiotic *Lactobacillus reuteri* strain^17^. Many lactobacilli species are known resident gut bacteria^18^. Dietary prebiotics are known to influence growth and metabolism of gut microorganisms and have positive effect on host health^19^. Fructan based prebiotics such as fructooligosaccharides and inulin are known to influence growth and metabolism of lactobacilli species as well as multiple other species of gut microorganisms such as *Bifidobacterium*, *Faecalibacterium*, *Bacteroides*^20,21^. Therefore, we explored the ability of two key prebiotics inulin and short-chain FOS (scFOS) to enhance the biosynthesis of precursor of vitamin D_3_ through metabolic route.

A comprehensive metabolic model would be suitable to investigate the interactions between these small molecules, microorganisms, and human metabolism. Such model would allow mechanistic understanding of the metabolic effects of prebiotic supplementation. Flux balance analysis (FBA) is a constraint-based modeling approach that allows analysis of such comprehensive metabolic models^22^. These metabolic models contain networks of all known metabolic reactions. FBA allows calculation of flux or rate of metabolite flow through the reaction network. This enables prediction of biologically relevant processes such as growth rate or metabolite synthesis rate. FBA is well suited for investigating the effects of external conditions such as diet, and optimizing relevant objective functions such as metabolite production or health condition. This approach has been used for studying metabolism of individual gut microorganisms and microbial community^23,24^. Further, a study of human and gut microorganisms including probiotics^25^ has reported the effect of gut microorganisms on human metabolites under different dietary conditions. However, to the best of our knowledge, detailed assessment of the effect of prebiotics on human metabolism with mechanistic details is not yet reported.

In the current work, we used co-metabolism models of human and gut microorganisms to investigate the effect of prebiotics for increasing vitamin D_3_ through its precursor 7-DHC. It is known that most common gut microorganisms belong to genera *Bacteroides*, *Faecalibacterium*, *Clostridium*, and *Eubacterium*^26^. Our previous analysis has also shown the relative abundance of each *Faecalibacterium* and *Bacteroides* to be about 0.1^27^. Therefore, as an initial screen, we investigated the impact of prebiotic supplementation through two such common microorganisms, *Faecalibacterium prausnitzii* and *Bacteroides thetaiotamicron*. We investigated the underlying metabolic reaction network to understand the mechanism and observed that prebiotics resulted in increased secretion of microbial metabolites such as lactate, acetate, and pyruvate. These metabolites are absorbed by human cells and lead to increased biosynthesis of 7-DHC. Interestingly, we observed organism and prebiotic specific differences in the profile of secreted metabolites as well as distinct dose dependent optimal flux modes for different prebiotics. Extending the analysis to 119 gut microorganisms having capability of utilizing at least one of the prebiotics, we observed that majority of these gut microorganisms respond favorably to prebiotic supplementation in terms of secretion of acetate, lactate or pyruvate. These bacteria can therefore facilitate increase of 7-DHC flux.

In summary, the current study shows the potential of prebiotics supplementation for increasing 7-DHC i.e. provitamin D_3_ biosynthesis flux. The study suggests a novel hypothesis for further experimental assessment of vitamin D levels with prebiotics supplementation.

## Results

We assessed the impact of prebiotic supplementation on the intermediates of vitamin D_3_ biosynthesis pathway using human-gut microorganism co-metabolism models representing vitamin D_3_ deficient conditions. We examined two widely used prebiotics, inulin and scFOS for each human-*Faecalibacterium prausnitzii* (hFP) and human-*Bacteroides thetaiotamicron* (hBT) models. Attributes of the model are summarized in supplementary information (SI). In order to represent vitamin D_3_ deficient conditions, we considered maximization of 25-hydroxyvitamin D_3_ secretion as the objective function. This molecule is considered as a biomarker associated with vitamin D_3_ status of the body and also represents one of the endpoints in the biosynthetic pathway.

### Effect of prebiotic supplementation on 7-DHC flux

**Figure 1:**
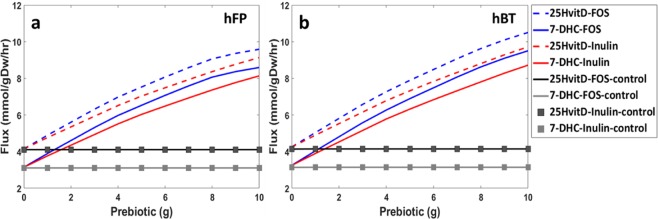
Effect of prebiotics on 7-DHC flux. Plots showing flux of 7-DHC synthesis (solid line) and 25-hydroxyvitamin D_3_ (dashed line) reaction for inulin (red) and scFOS (blue) supplementation for (**a**) hFP and (**b**) hBT model. Black and grey lines with marker are control where, fluxes through secretion reactions from microorganisms are limited.

Vitamin D_3_ is synthesized from 7-DHC spontaneously on exposure to UVB and heat. Therefore, as a final step in the enzymatic biosynthesis process, we examined the effect of prebiotic supplementation on 7-DHC synthesis. We investigated the impact of prebiotics supplementation by varying the prebiotic uptake fluxes corresponding to a range of 0 to 10 g, as 10 g is the commonly accepted safe dose of scFOS. We observed dose dependent increase in 7-DHC flux and a corresponding increase in 25-hydroxyvitamin D_3_ secretion reaction flux (Fig. 1). Both hFP and hBT models showed qualitatively similar response to prebiotic supplementation. We observed more than 2 fold increase in the flux of 7-DHC and 25-hydroxyvitamin D_3_ secretion for uptake dose of 10 g.

Both hFP and hBT models showed 5% and 9% higher flux of 7-DHC respectively, for scFOS supplementation as compared to inulin. Additionally, in comparison to hFP, hBT exhibited 10% and 7% higher flux of 7-DHC for scFOS and inulin respectively.

Interestingly, when the secretion fluxes were computed restricting the microorganisms’ exchange reactions flux to a limiting value such that the system remains viable; no changes in the flux of 7-DHC and 25-hydroxyvitamin D_3_ were observed (Fig. 1). This confirmed the significance of gut microbial metabolism in affecting 7-DHC biosynthesis flux in human metabolism.

### Comparative assessment of organism specific mechanisms for flux enhancement

**Figure 2:**
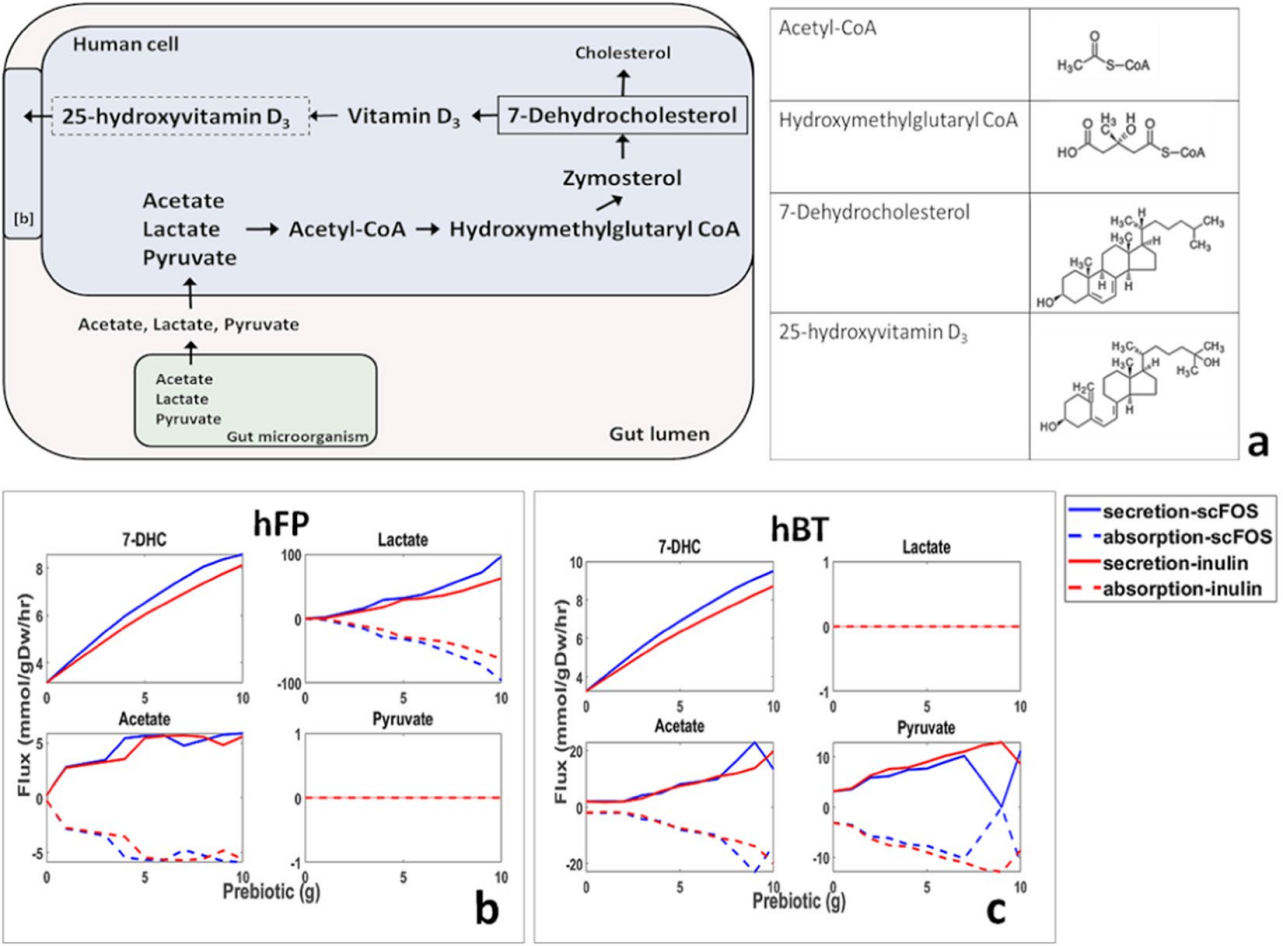
Effect of prebiotics on metabolite secretion and absorption. (**a**) Schematic representation of biochemical pathway; [**b**] represents human body compartment. Prebiotics dose dependent changes in secretion (solid line) and absorption (dashed line) fluxes for supplementation of inulin (red) and scFOS (blue) For (**b**) hFP and (**c**) hBT model.

In order to investigate the relationship between prebiotics, gut microbial metabolism and human metabolism leading to increased 7-DHC, we identified a reaction pathway in the co-metabolism models by comparative analysis of reaction fluxes. A schematic biochemical pathway indicating the synthesis of vitamin D_3_ is represented in Fig. 2a. The flux was observed to flow from hydroxymethylglutaryl CoA resulting in 7-DHC, a branch point for synthesis of vitamin D_3_ and cholesterol. Hydroxymethylglutaryl CoA, in turn, is synthesized from acetoacetyl-CoA and acetyl-CoA. We identified each of the exchange reactions from hFP and hBT models reporting flux changes with prebiotic supplementation. We further investigated the microbial exchange reactions for secretion fluxes and examined corresponding exchange reactions in human metabolic network showing increase in absorption fluxes. We identified acetate, lactate and pyruvate as key metabolites resulting in synthesis of acetyl-CoA. This in turn influenced the flux through acetoacetyl-CoA and impacted 7-DHC flux. Figures 2b,c show dose dependent changes in exchange fluxes of acetate, lactate and pyruvate for the two models.

We observed that lactate secretion and corresponding absorption flux was about 20 times as that of acetate for hFP model, while lactate was not secreted in case of hBT. Interestingly, the opposite scenario was observed in case of pyruvate, where it was secreted in hBT but not in hFP. This indicated that there were organism-specific metabolites that affect the synthesis of acetyl-CoA, which eventually participates in the synthesis of 7-DHC. In order to identify the impact of each of the metabolites on 7-DHC, we switched off the causal reactions one at a time and examined the effect on 7-DHC flux. As expected, we observed readjustment of fluxes of other metabolite exchange reactions in order to maximize the flux of 25-hydroxyvitamin D_3_ biosynthesis. We observed that in case of hFP model, switching off lactate exchange reaction resulted in about 10% reduction in 7-DHC flux. In case of hBT a single exclusive metabolite having large impact was not observed however considerable changes in the fluxes of other metabolites were observed (Supplementary Figures S1 and S2). From Fig. 2b, it was clear that in case of hFP the exchange flux through lactate was considerably higher compared to the other two metabolites. Therefore, switching off lactate exchange affected flux through 7-DHC. In case of hBT, no single metabolite reaction with considerably high exchange flux was observed (Fig. 2c). Thus switching off single reaction did not affect the flux through 7-DHC.

### Screen for prebiotic dependent metabolite secretion by gut microorganisms

**Figure 3:**
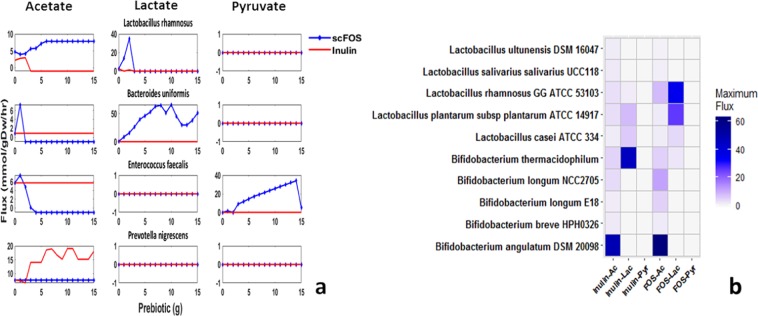
Organism specific response to prebiotics. (**a**) Dose dependent acetate, lactate and pyruvate secretion profiles by gut microorganisms for inulin (red) and scFOS (blue) (**b**) Heatmap of maximum flux for acetate, lactate and pyruvate for inulin and scFOS.

With the identification of microbial metabolites affecting 7-DHC, we hypothesized that gut microorganisms capable of metabolizing these prebiotics and secreting any of these metabolites would be helpful in increasing 7-DHC biosynthesis flux. Therefore, as a next step, we assessed 818 available genome scale metabolic models of gut microorganisms for their ability to utilize either inulin or scFOS. We identified 119 organism models that are reported to utilize either or both the prebiotics. Of these, 20 organisms were observed to utilize both inulin and scFOS while 54 and 45 organisms were observed to utilize scFOS and inulin alone, respectively. We investigated the response of these gut microorganisms in terms of secretion of acetate, lactate and pyruvate. Figure 3a shows secretion flux profiles of these metabolites with prebiotic supplementation for four organisms from different genera as an example. The complete dose dependent response for all the 119 organisms is given in Supplementary Figure S3. In order to comparatively assess the efficacy of prebiotics, we also examined the maximum extent to which these metabolites are secreted. Figure 3b shows maximum value for metabolite flux with prebiotic supplementation within a range of 0–10 g, for some species of *Lactobacillus* and *Bifidobacterium* as an example. The response for all the 119 organisms is given in Supplementary Fig. S4.

We observed differential response of these organisms to inulin and scFOS in terms of both the secreted metabolites and also the extent of secretion flux (Fig. 3a,b). Nine organisms did not secrete any of these metabolites. These organisms were mainly from genus *Clostridium* and *Coprococcus*. Rest of the organisms secreted acetate at the least. Interestingly, organisms were observed to either secrete lactate or pyruvate. We observed that 27 organisms secreted both acetate and lactate. These organisms belonged mainly to *Lactobacillus*, *Prevotella* and *Bacteroides* genera. Interestingly, *Prevotella* species were observed to produce high level of lactate with inulin supplementation, while *Bacteroides* species were observed to do the same with FOS supplementation. It was observed that pyruvate was secreted by few organisms. Only five organisms, mainly belonging to *Enterococcus* genus, were observed to secrete acetate and pyruvate.

We observed variation in the extent to which a metabolite was secreted within species of same genus. For instance, from Fig. 3b we observed that *Bifidobacterium angulatum* secretes higher level of acetate as compared to other species. Similarly, *Lactobacillus rhamnosus* and *Lactobacillus planterum* secrete higher levels of lactate as compared to other species of *Lactobacillus*. Overall, microorganisms from genera such as *Bacillus*, and *Bacteroides* were observed to produce higher level of acetate for both prebiotics.

### Flux modes dependency on prebiotic dosage

**Figure 4:**
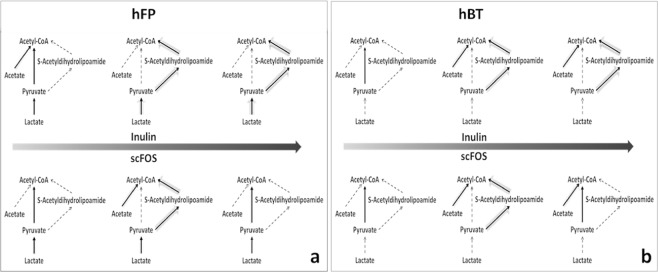
Flux modes in reaction network. Flux modes for 1 g, 3 g and 5 g dose of prebiotics for (**a**) hFP and (**b**) hBT. Dashed arrow represents zero flux through the reaction. Thickness of arrow represents the extent of flux through the reaction.

Organism specific and dose dependent variation in secretion fluxes of certain metabolites led us to investigate the distribution of flux modes in the underlying reaction network. We delineated reaction pathways in host from acetate, lactate, and pyruvate leading primarily to acetyl-CoA which eventually contributes to synthesis of 7-DHC. We investigated flux through these specific reactions for both the prebiotics as a function of prebiotic dosage. Figure 4 summarizes the flux modes observed in human metabolic network for the two models, at 1 g, 3 g and 5 g as representative dose levels of the prebiotics.

In general, acetate was converted to acetyl-CoA at higher prebiotic doses in case of hBT model (Fig. 4b) while flux was mainly directed through pyruvate in case of hFP (Fig. 4a). These flux modes are influenced by the organism specific availability of primary metabolites.

As maximization of 25-hydroxyvitamin D_3_ secretion was achieved by readjusting the fluxes of reactions in the metabolic network, we examined the fluxes of all the internal human metabolic reactions. We scrutinized top 90% of the reactions based on percent relative flux change for single dose of prebiotics (i.e. 5 g). As expected, a large fraction of reactions from biochemical subsystems such as fatty acid metabolism, cholesterol biosynthesis, squalene and cholesterol synthesis, oxidative phosphorylation, pentose phosphate pathway showed changes in flux values. Additionally, reactions belonging to bile acid synthesis, folate metabolism, certain amino acid metabolism also showed changes in the flux values. This observation was consistent across the models and prebiotics. The details of this analysis are given in the supplementary information (SI).

## Discussion

The study shows that supplementation of prebiotics can help increase 7-DHC biosynthesis flux. In addition to external factors such as latitude, season, clothing habits and application of sunscreen that decrease the exposure to UVB, reduced precursor is an important intrinsic factor for vitamin D_3_ deficiency. Age and burn injuries are reported to result in considerable reduction of 7-DHC^7^. Additionally, skin melanin content competes with 7-DHC for UVB absorption. In such cases, increasing 7-DHC can result in increased cutaneous synthesis of vitamin D_3_. However, the overall vitamin D status in an individual is determined by multiple extrinsic and intrinsic factors such dietary habits, use of sunscreen, baseline vitamin D values and is known to vary widely in global populations^28,29^. Increasing the levels of 7-DHC, a critical intermediary metabolite in the biosynthetic process, through prebiotic supplementation could assist in dealing with vitamin D deficiency.

Probiotics, in addition to multiple health benefits, are known to have positive effects in alleviating vitamin D_3_ deficiency. Since prebiotics facilitate growth and metabolism of probiotics and other resident gut microorganisms, these are good candidates to deploy for this effort. We examined two important fructan prebiotics viz., inulin and scFOS to increase the biosynthesis of 7-DHC mediated through the activity of common gut microorganisms. A two fold increase in 7-DHC synthesis flux indicated the effectiveness of prebiotics supplementation. Knowledge of initial concentration, however, would be required to predict the absolute level of 7-DHC. For both the hFP and hBT models, we observed scFOS supplementation showing higher flux compared to inulin supplementation.

Use of comprehensive genome scale co-metabolism model enabled us to investigate the mechanistic details of the effect of prebiotic supplementation. In this study we have adapted previously developed genome scale models of both the microorganisms and the human host. Given the current network structure and the constraints we observed around two fold increase in 7-DHC biosynthesis flux in response to prebiotic supplementation. As the underlying models are refined the observed response may vary depending upon the changes to the network connectivity or the constraints. We observed that there were organism specific, prebiotic dependent variations in the secretion of acetate, lactate and pyruvate. These metabolites were absorbed in the host eventually leading to synthesis of 7-DHC. A clinical trial analysis by Prakash and co-workers^17^ reported that oral supplementation of probiotic strain *Lactobacillus reuteri* NCIMB 30242 resulted in significant increase of serum 25-hydroxyvitamin D_3_ level. The study has proposed increased lactic acid production, and increased 7-DHC synthesis as potential mechanisms responsible for this effect. In coherence, our simulation showed lactate as one of the metabolites leading to increased 7-DHC flux. Switching off lactate secretion led to decrease in the flux value. Our assessment of gut microorganisms also showed increased lactate secretion for prebiotic supplementation by multiple *Lactobacillus* species. Additionally, we observed other metabolites such as acetate and pyruvate that can be directed for biosynthesis of 7-DHC. The screening showed different metabolite secretion profiles for gut microorganisms for the two prebiotics tested. Thus depending upon gut microbial content of an individual there can be differential response to prebiotic supplementation. This screening study can serve as a starting point for investigating the effect of prebiotics on other microbial metabolites beneficial to the host. We observed multiple flux modes as a function of prebiotic dose. This indicated multiple potential routes for directing the metabolites depending upon availability of the substrates. We observed two regimens of flux modes. At lower prebiotic dose and corresponding lower metabolite availability, acetyl-coA was synthesized from pyruvate through decarboxylase reaction, simultaneously generating NADH that can be used to generate ATP. On the other hand, at relatively higher prebiotic dose and corresponding higher availability of metabolites, acetyl-CoA was synthesized either through S-Acetyldihydrolipoamide reaction or from acetate that required ATP. Therefore, depending upon the availability of metabolites, distinct flux modes were observed to be optimal in order to maximize 25-hydroxyvitamin D_3_ flux. Overall, the analyses showed that there were organism specific and prebiotic dependent secretion of certain metabolites that were directed for biosynthesis of 7-DHC. This provides testable hypotheses for further investigation.

Basic FBA framework considers the stoichiometric and thermodynamic constraints and does not take into account regulatory aspects such as transcriptional, post-transcriptional, post-translational regulation. Thus the current model analyses indicated the potential to direct the metabolites obtained from microorganism for biosynthesis of 7-DHC. Unless specific constraints are defined, the current framework assumes that all the enzymes in the reaction pathways are available and are in active state. Thus to understand variability in the population, responses of multiple condition specific models need to be considered. Given the current focus to analyze the metabolic potential of prebiotic supplementation, this framework seems sufficient to provide the details of biochemical mechanism. Addition of other regulatory aspects using approaches like rFBA, dynamic FBA would refine the response for prebiotic supplementation. Given that other biochemical parameters remain same for with and without supplementation conditions, increase in biosynthesis flux can lead to increase in metabolite level. In the simulations, we observed saturation of 7-DHC biosynthesis flux beyond certain prebiotics dose. This indicated inherent regulation imposed due to thermodynamic constraints and network connectivity. Therefore, 7-DHC level would not increase above a certain threshold level for any given dose of prebiotics. As a limitation, the current framework considers only the rates of reactions and not metabolite concentrations. Knowledge of initial metabolite concentration would enable prediction of final concentration based on the flux obtained from the simulations. Given the steady state approach there is no consideration of the time required to attain the observed rate of biosynthesis. The knowledge of these time scales is an important factor for consideration in dynamic biological cellular systems. In addition to consideration of reaction kinetics, multiple compartment transport, accumulation and depletion kinetics of metabolites is required. Specifically, accumulation and depletion kinetics for attaining specific physiological target level need to be further investigated^30^.

One crucial factor influencing the effect of prebiotic is the composition of gut microbiome. In the current study, we examined the effect of prebiotics through co-metabolism with *F*. *prausnitzii* and *B*. *thetaiotamicron* as representatives of common genera of gut microorganisms. Additionally, we also examined the response of 119 microorganisms for metabolite secretion profile with prebiotic supplementation and observed differential response. These results indicated that, depending upon the composition of gut microbiome, prebiotic supplementation would result into distinct metabolite outputs. This observation is of particular importance in prioritizing the use of prebiotics depending upon the gut microbiome composition. Majority of the organisms were observed to secrete acetate, while lactate and pyruvate were observed to be secreted by certain genera of organisms. Studies have shown that individuals can be grouped based on variation in the levels of dominant genera viz., *Bacteroides*, and *Prevotella* in their microbiome^31^. Our observation of preferential secretion of lactate by *Bacteroides* and *Prevotella* species for scFOS and inulin respectively, is useful in selecting a preferred prebiotic supplementation.

Depending upon factors such as geography, diet, age and gender, the composition of gut microbiome is known to vary considerably. Changes in total and relative abundance of microorganisms would result in changes in the effective availability of prebiotics to the gut microorganisms. Thus, the observations of this study represent a potential response to prebiotics supplementation. To simulate such conditions we have performed dose-response computations. The metabolite profiles were consistent in the range of prebiotic doses. Additionally, knowing the gut microbial composition and the relative abundance of gut microorganisms, preferred prebiotic and the dose of prebiotic can be determined using the modeling framework. Additional factors such as use of drugs or certain clinical conditions would affect individual’s response to prebiotics supplementation. Use of drugs such as antibiotics that severely affect microbial population or clinical conditions that lead to acute dysbiosis of intestinal microbiome would have an effect on the response to prebiotics supplementation.

As a part of screening gut microorganisms, some of the probiotic organisms such as *Lactobacillus* and *Bifidobacterium* species were examined for their response to prebiotics. This has an implication in development of synbiotics. With detailed genome scale metabolic models of gut microorganisms this investigation can further be extended to screen for various classes of prebiotics for their effect on bacterial growth and metabolites. Dietary prebiotics are known to provide multiple health benefits to the host. Our study suggests a distinct potential application for the use of prebiotics in reducing vitamin D_3_ deficiency caused due to reduced 7-DHC.

## Methods

### Development of condition specific Human and gut microorganism co-metabolism model

We used the genome scale co-metabolism model of human - *F*. *prausnitzii* (hFP) and human – *B*. *thetaiotamicron* (hBT) developed previously^25^. The compartment structure of the co-metabolism model is represented in Supplementary Fig. S6. In brief, the co-metabolism models contain two separate genome scale metabolism reaction network models that are joined through a common lumen compartment. The food components enter the lumen compartment, through metabolite exchange reactions. These metabolites can be taken as input by human and/or microorganism depending on their respective metabolic capabilities.

Condition specific model is developed by imposing constraints on reaction flux bounds to represent certain nutrient uptake. The developed models consider western dietary conditions^25^. Prebiotic supplementation is simulated as additional constraints on the corresponding exchange reactions. Inulin supplementation is simulated by constraining the lower bound of inulin exchange reaction to represent absorption of inulin. scFOS comprises of three components, kestose (GF2), kestotetraose (GF3) and kestopentaose (GF4), based on fructose chain length. To simulate scFOS supplementation, lower bound of exchange fluxes of all the three reactions is constrained representing absorption. For both the prebiotics, the fluxes are varied in a range corresponding to 0 g to 10 g dose. Values for flux range are given in supplementary information.

### Screening of gut microorganisms for prebiotic effect

Genome scale models for 818 gut microorganisms were obtained from VHM database^32^ having western diet constraints. Prebiotic supplementation is implemented as mentioned previously. The prebiotic dose is varied in the range of 0–10 g. The maximum flux value in this range for each of the three metabolites is plotted in the heatmap.

### Simulation of vitamin D_3_ deficiency condition

Under limiting or deficient conditions for a certain component, a biological system would try to synthesize or obtain the component by compensating the fluxes through its reaction network in order to overcome the stress. Therefore, it would be appropriate to consider maximization of vitamin D as the objective under vitamin D deficient conditions. As serum 25-hydroxyvitamin D_3_ is considered as an indicator of vitamin D level, in this case, to simulate vitamin D_3_ deficiency we defined 25-hydroxyvitamin D_3_ secretion maximization as the objective function. We compared the fluxes for conditions of basal diet without and with supplementation of prebiotics.

### Simulation of reaction knock-off

In order to investigate the extent of effect of each of the key metabolite secreted from microbe compartment, we simulated single reaction knock-off. For reaction knock-off simulation both the lower and upper bound of corresponding secretion reaction flux are constrained to zero. Other dietary constraints and the objective function are kept unchanged.

### Subsystem distribution analysis

For each of the co-metabolism model, flux values are compared for without and with 5 g supplementation of each of the prebiotics. We calculated percent relative change in flux for all internal metabolism reactions of human compartment. Top 90% reactions showing change are selected for subsystem analysis. The fraction is calculated as the ratio of reactions of a biochemical subsystem with flux change to the total number of reactions of that subsystem and is given as,

$$subsystem\,fraction=\frac{number\,of\,reactions\,in\,subsystem\,with\,flux\,change}{total\,reactions\,of\,the\,subsystem}$$

All the simulations were performed in Matlab 2018a, The MathWorks, Inc., Natick, Massachusetts, United States, using COBRA toolbox^33^. Analyses of fluxes were performed using in-house scripts written in R and Perl.

## Supplementary information

Supplementary Information

Scripts
