# Supplementary material for: Provitamin D3 modulation through prebiotics supplementation: simulation based assessment
Source: Sci Rep. 2019 Dec 17;9:19267. doi: 10.1038/s41598-019-55699-2 (PMC6917722; doi:10.1038/s41598-019-55699-2)
Supplement: Supplementary file 1 — Supplementary Information [file 41598_2019_55699_MOESM1_ESM.docx]

# Provitamin D_3_ modulation through prebiotics supplementation: simulation based assessment

Sucheta Gokhale* and Anirban Bhaduri

**Supplementary Information**

Details of hFP and hBT model

|  | Human model compartment | FP model compartment | BT model compartment |
| --- | --- | --- | --- |
| Total reactions | 9894 | 1030 | 1528 |
| Internal metabolism reactions | 8575 | 880 | 1248 |
| Exchange reactions | 661 | 150 | 280 |
| Body fluid exchange | 658 | - | - |

*Subsystem distribution of reactions with altered flux values*

We analyzed the flux values at prebiotic exchange flux equivalent of 5g dose. To identify the extent to which other biochemical systems are affected we investigated top 90% of the reactions based on percent relative flux change. Figure S5 shows the fraction of reactions of biochemical subsystems with flux changes for inulin and scFOS supplementation for both the organisms. We observed that for both the prebiotics and in case of both the organisms overall subsystem distribution and reaction fractions were similar.

Reactions from subsystems fatty acid metabolism, cholesterol biosynthesis, squalene and cholesterol synthesis, oxidative phosphorylation, pentose phosphate pathway showed change in flux values. Additionally, reactions belonging to bile acid synthesis, folate metabolism, certain amino acid metabolism also showed changes in the flux values. We observed that in case of co-metabolism with *F. prausnitzii*, more than half of the reactions of vitamin B2 metabolism showed flux changes. We looked at those specific reactions and found that these reactions mainly represent cofactor FMN and FAD conversion driven by ATP, and synthesis of vitamin B2 is not affected.

As the biosynthesis pathway for vitamin D_3_ and cholesterol biosynthesis shares common reactions, we also examined the levels of cholesterol. Under these conditions, we did not observe any increase in cholesterol level with prebiotic supplementation.

*Flux value ranges for prebiotic supplementation*

The ranges for lower flux bound equivalent to 0 to 10 g dose for exchange reactions

For EX_kesto reaction: 0 to -9.1191 mmol gDW^-1^hr^-1^

For EX_kestottr reaction: 0 to -6.7509 mmol gDW^-1^hr^-1^

For EX_kestopt reaction: 0 to -0.9653 mmol gD W^-1^hr^-1^

For EX_inulin reaction: 0 to -1.6183 mmol gD W^-1^hr^-1^

Upper flux bound of these reactions and other dietary constraints are kept unchanged.

**Supplementary Figures**


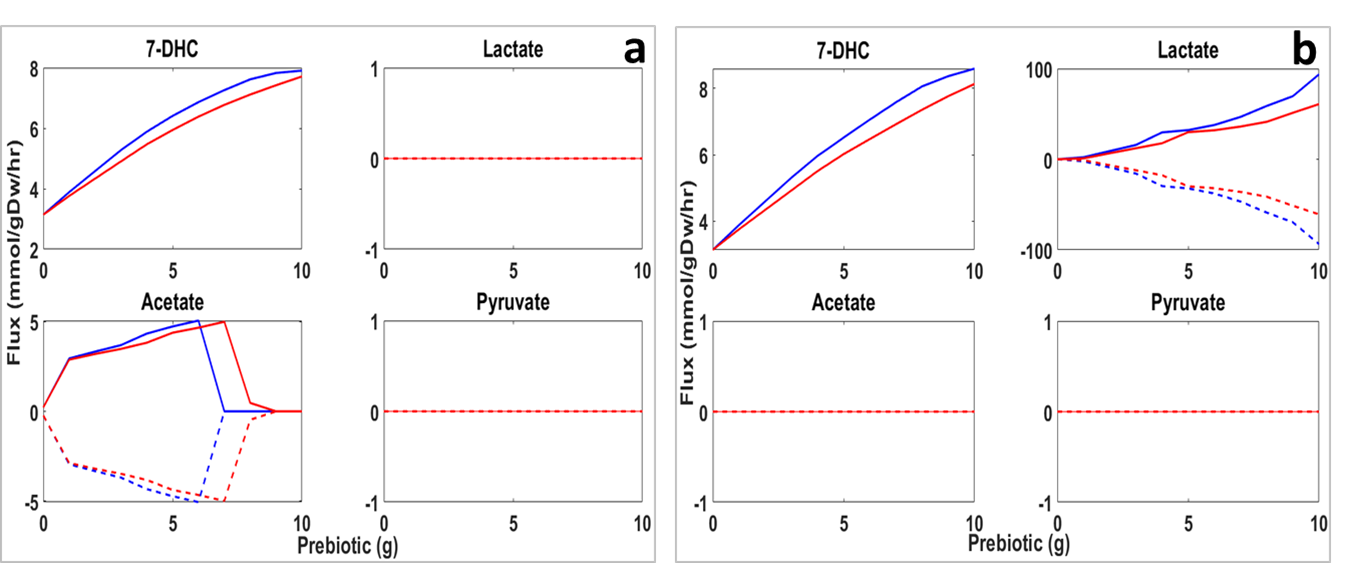


Figure S1: Prebiotic dose dependent changes in secretion fluxes (solid line) and absorption fluxes (dashed line) for supplementation of inulin (red) and scFOS (blue) for co-metabolism with *F. prausnitzii* with reaction knock-off for (a) lactate (b) acetate.

Pyruvate secretion reaction is absent in *F. prausnitzii*.


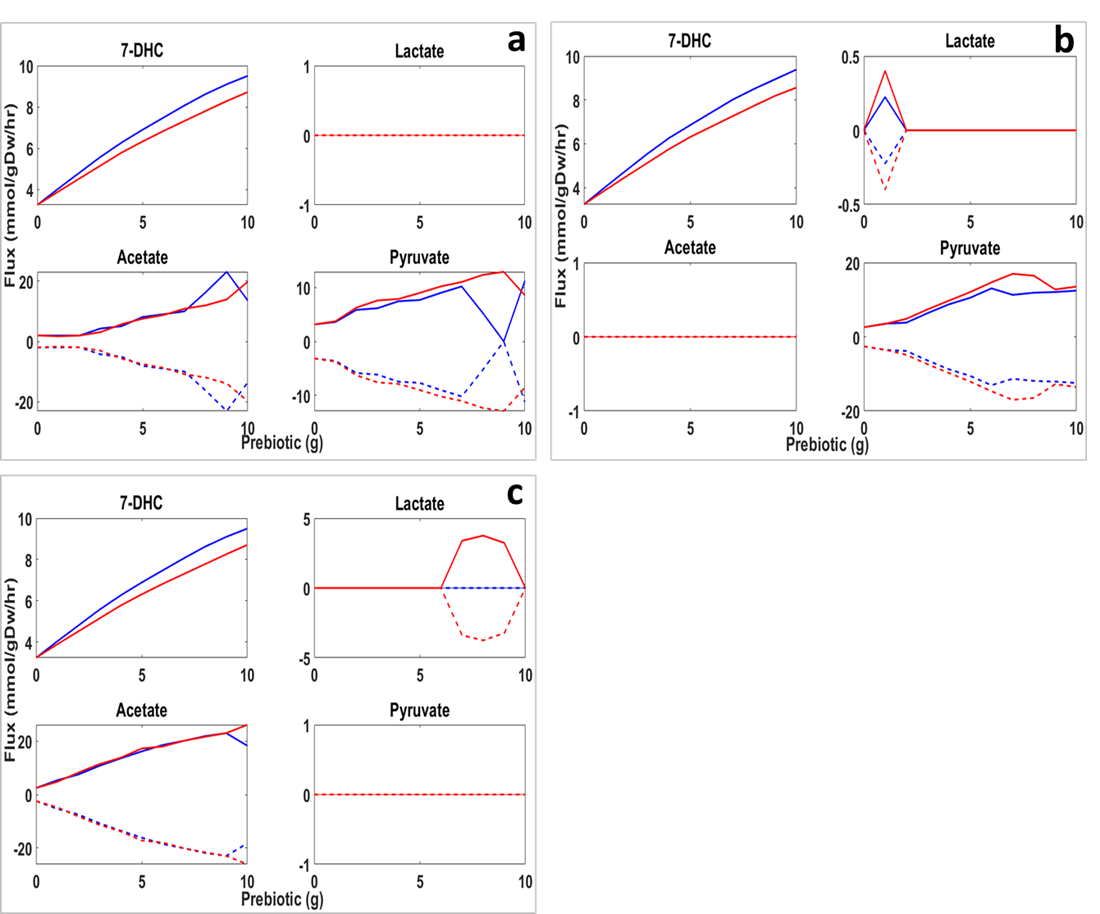


Figure S2: Prebiotic dose dependent changes in secretion fluxes (solid line) and absorption fluxes (dashed line) for supplementation of inulin (red) and scFOS (blue) for co-metabolism with *B. thetaiotamicron* with reaction knock-off for (a) lactate (b) acetate (c) pyruvate.


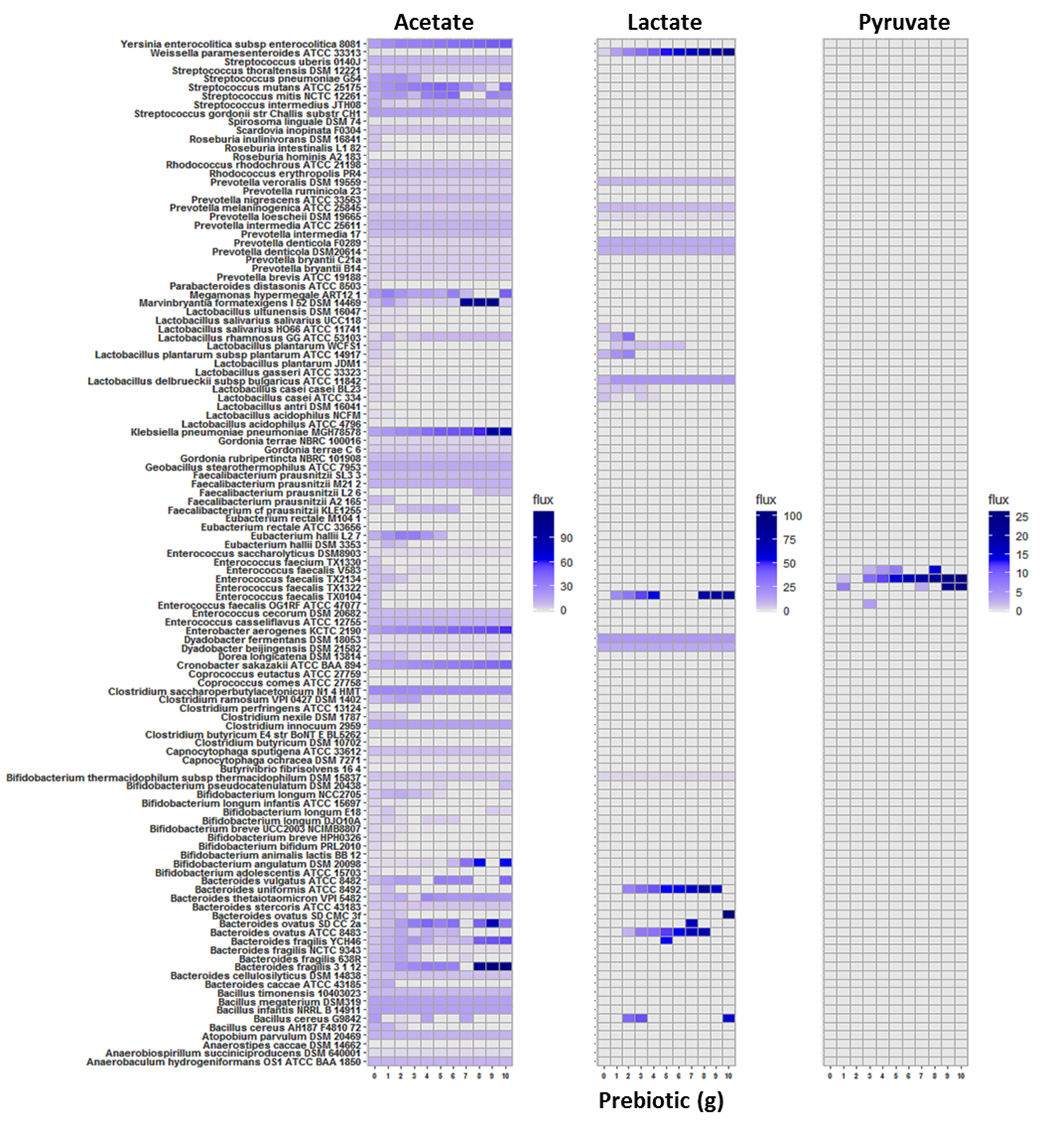


Figure S3a: Heatmaps showing scFOS dose dependent flux for acetate, lactate and pyruvate for 119 gut microorganisms


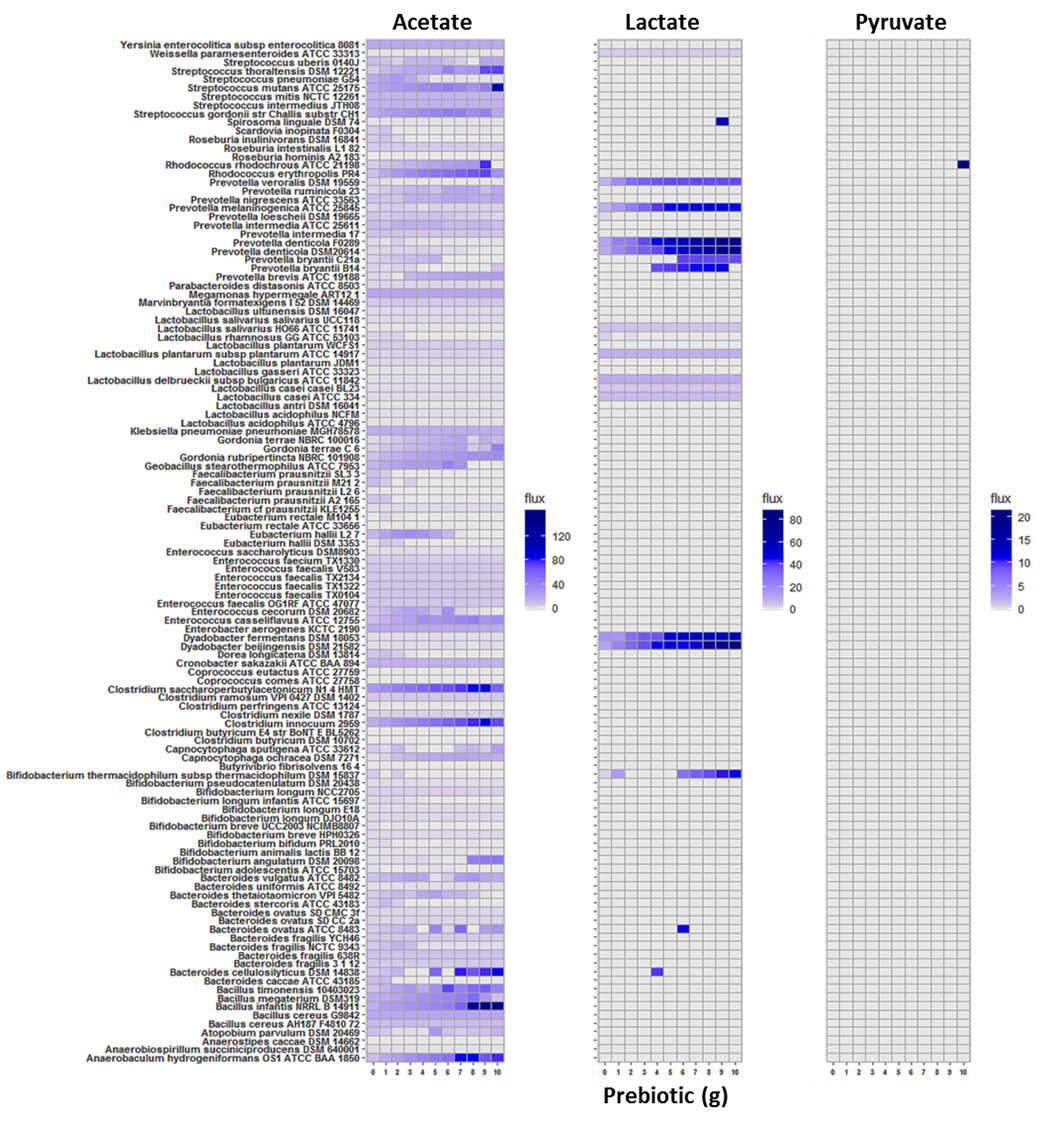


Figure S3b: Heatmaps showing Inulin dose dependent flux for acetate, lactate and pyruvate for 119 gut microorganisms


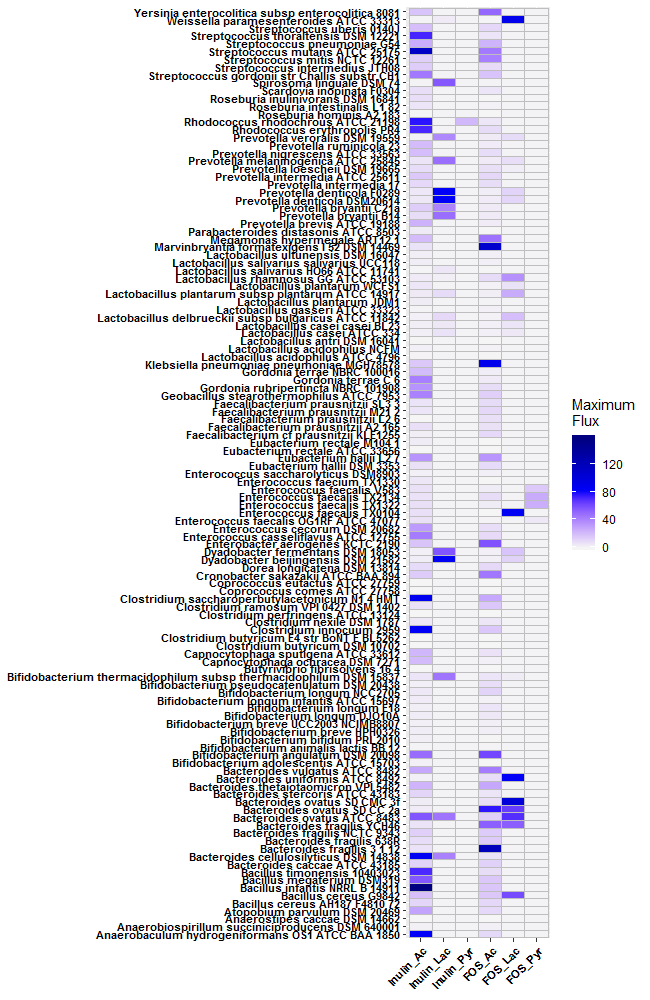


Figure S4: Heatmap of maximum flux for organisms for acetate, lactate and pyruvate for scFOS and inulin


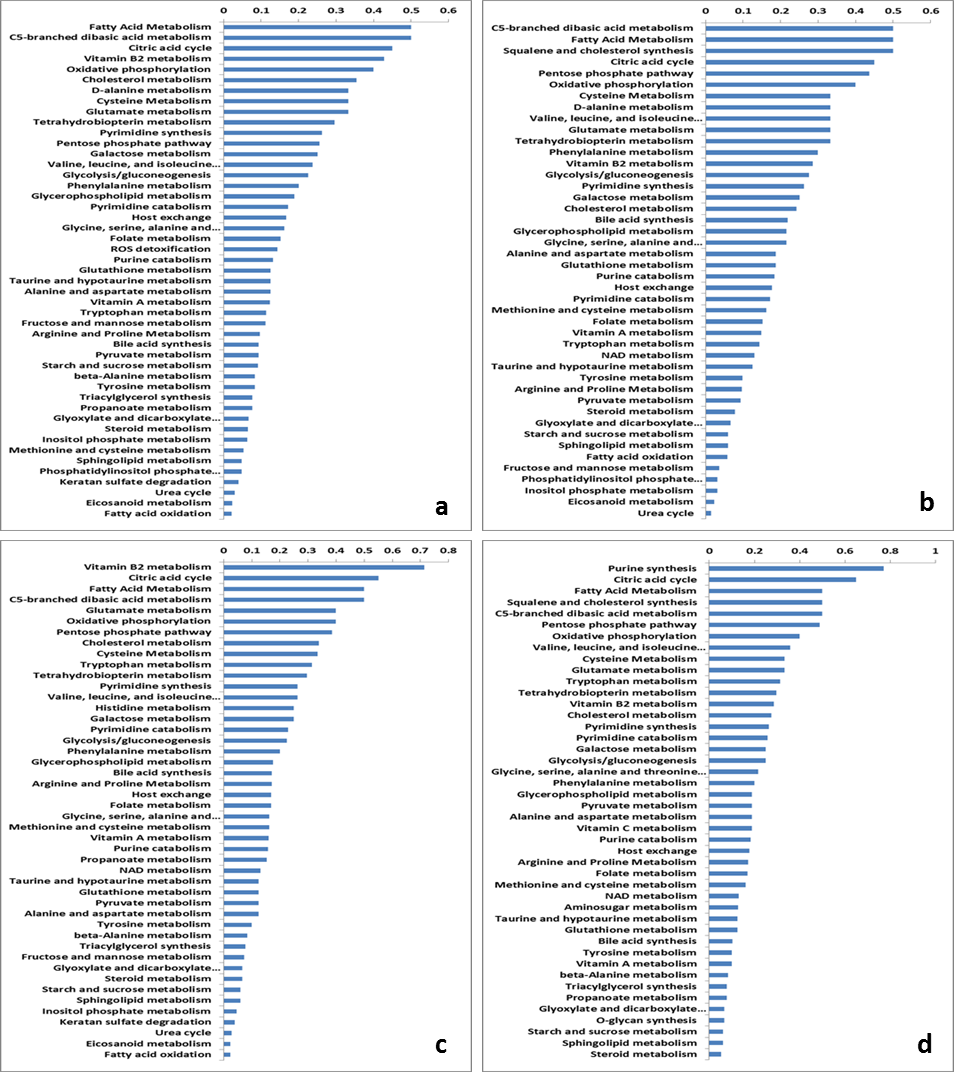


Figure S5: Reaction subsystem distribution. Fraction of reactions of metabolic subsystems showing flux change with prebiotic supplementation (a) and (b) for inulin, (c) and (d) for scFOS. Co-metabolism with *F. prausnitzii* (a) and (c), *B. thetaiotamicron* (b) and (d).


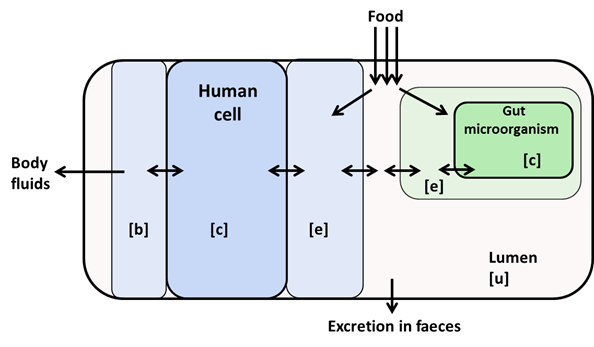


Figure S6: Schematic representation of compartment co-metabolism model. [c] – cellular compartment, [e] – extracellular compartment, [u] – lumen compartment, [b] – body compartment. Arrows represent metabolite exchange between compartments.
